# F151. A SYSTEMATIC REVIEW OF TASK-BASED FUNCTIONAL NEUROIMAGING STUDIES IN THOUGHT DISORDER

**DOI:** 10.1093/schbul/sby017.682

**Published:** 2018-04-01

**Authors:** Philip Sumner, Imogen Bell, Susan Rossell

**Affiliations:** 1 Swinburne University of Technology; 2 Monash Alfred Psychiatry Research Centre, The Alfred Hospital and Monash University, Central Clinical School, Swinburne University of Technology, The University of Melbourne, St. Vincent’s Hospital

## Abstract

**Background:**

Thought disorder (TD) is an important symptom that demonstrates familial aggregation, predicts conversion to psychosis in those at risk, and predicts the duration and rate of hospitalisation in those with psychosis. However, the aetiology of TD is debated, with theoretical accounts revolving around executive, language, and semantic impairments. The aim of the current systematic review was to synthesise the research that has investigated TD using task-based functional neuroimaging techniques to target executive, language, or semantic functions.

**Methods:**

The literature search was conducted in accordance with the Preferred Reporting Items for Systematic Reviews and Meta-Analyses (PRISMA) statement. The databases: PubMed, Scopus, and Web of Science were used to locate relevant literature from January 1990 to August 2016. The search strategy was broad and inclusive to capture exploratory and secondary TD-related analyses.

**Results:**

The search yielded 5821 records, from which 37 pertinent studies were identified. Functional correlates of TD included the superior and middle temporal, fusiform, and inferior frontal gyri bilaterally, as well as the left and right cingulate cortex, the right caudate nucleus, and the cerebellum. TD-related increases and decreases in activation were both evident in most of these regions. However, the specificity of these correlates from general clinical and cognitive influences, as well as the relationships between task-based function and behavioural performance, are currently unknown.

**Discussion:**

The cortical regions implicated overlap with those thought to contribute to language and semantic systems. Cortico-striatal circuitry may additionally play a role in some aspects of TD through aberrant salience representation and inappropriate attentional prioritisation. To advance the field further, greater integration across structural, functional, and behavioural measures is required, in addition to non-unitary considerations of TD and more thorough investigations of component cognitive processes.

